# Evaluation of Respiratory Symptoms among Workers in an Automobile Manufacturing Factory, Iran

**Published:** 2018-02

**Authors:** Bahram HARATI, Seyed Jamaleddin SHAHTAHERI, Ali KARIMI, Kamal AZAM, Alireza AHMADI, Maryam AFZALI RAD, Ali HARATI

**Affiliations:** 1. Dept. of Occupational Health Engineering, School of Public Health, Tehran University of Medical Sciences, Tehran, Iran; 2. Dept. of Environmental Chemical Pollutants and Pesticides, Institute for Environmental Research, Tehran University of Medical Sciences, Tehran, Iran; 3. Dept. of Epidemiology and Biostatistics, School of Public Health, Tehran University of Medical Sciences, Tehran, Iran; 4. Dept. of Occupational Health Engineering, School of Public Health, Shahid Beheshti University of Medical Sciences, Tehran, Iran; 5. Dept. of Occupational Health Engineering, School of Public Health, Islamic Azad University, Boroujerd Branch, Boroujerd, Iran

**Keywords:** Lung function, Respiratory symptoms, Dust, Silica, Compound BTEX

## Abstract

**Background:**

This study estimated the prevalence of respiratory symptoms and disorders among workers exposed to exposure to volatile organic compound (VOCs) in an automobile manufacturing factory in Tehran, Iran in 2016.

**Methods:**

Subjects of this case-control research were included 80 samples including 40 workers exposed to different level of BTEX as well as 40 unexposed individuals were considered as control group. Methods 1501 and 7602 presented by the National Institute of Occupational Safety and Health (NIOSH) were used for the sampling and analysis of compounds in the air. Gas Chromatography-Flame Ionization Detector (GC-FID) was used for analysis of compounds of interest. Six silica samples were collected during the campaign. Silica analyses were performed by using visible absorption spectrophotometry system. Lung functions were evaluated for 80 workers (40 exposed, 40 nonexposed) using spirometry system.

**Results:**

The average amount of total dust and free silica measured in factory were 7.3±1.04 mg.m^−3^ and 0.017±0.02 mg.m^−3^ respectively. Average benzene, toluene, ethyl-benzene and xylene exposure levels in exposed subject’s median were 0.775±0.12, 1.2±2.08, 45.8±8.5, and 42.5±23.9 ppm respectively. Statistical tests showed significant difference between pulmonary function tests (except PEF) of exposed and non-exposed individuals before and after employment (P<0.05). Workers exposed to VOCs presented lower levels of FVC, VC, and PEF than the control group except FEV1/FVC%, FEV1, FEF2575 and FEV1/VC%.

**Conclusion:**

Decline in lung volumes and respiratory symptoms, significant difference associated with the exposure to dust or gas, duration of exposure, and smoking habit. Therefore, lung function tests should be performed before and after the employment to identify sensitive workers candidates.

## Introduction

Exposure to different chemical substances and inhalation of dust in small and large scale industries may cause respiratory disorders in exposed workers (1, 2). Diseases of the respiratory system induced by occupational dust, gas, and vapors, are influenced by the type of dust, gas, vapor, and duration of exposure (3–5). Exposure to pollutant in the workplace may cause respiratory symptoms and disorders (6, 7). Residents and workers in the area are presumably experienced acute exposure to these contaminants as well as chronic exposure in and around homes and workplace during the following months (8). Some studies have shown numerous prevalence of respiratory symptoms in pollutants-exposed workers such as a chronic cough, dyspnea, chest tightness, rhinitis compared to the controls (9–11). Hanssen et al. reported the higher self-reported prevalence of chronic respiratory symptoms and dermal symptoms in workers inside the greenhouse, compared to the controls (12). Other potential respiratory health hazards can be caused by pesticides and tobacco smokes (13–15). Significant associations between smoking exposures and respiratory symptoms were observed (16). Whereas, lower association between respiratory symptoms and spirometry results was reported (8). There have been some reports on respiratory effects of dust exposure (17, 18).

Dust can cause respiratory problems for workers in the workplace as well as adverse effects on different organs such as lung, eyes, nose, and the airways (17).

Chemical substances products in the workplace have irritant and sensitizing effects on the airways and can induce respiratory disorders and can effect on lung function (19). Some disorders caused by volatile organic compounds (VOCs) have been determined such as hematologic effects, carcinogenic human health effects, harm to nervous system, toxic as well as increase of the global greenhouse effect. Benzene has shown to cause cancer in both animals and humans and therefore, is now classified by the Environmental Protection Agency (EPA) and the International Agency for Research on Cancer (IARC) as a human carcinogen (3, 20–22).

However, workers are potentially exposed to dust and toxic organic pollutants (PAHs, PTEX, and chlorinated hydrocarbons) that may have harmful effects on their respiratory health (18, 23). Despite numerous studies on respiratory disorders, few studies have considered average change in lung function measurements before and after employment in automobile manufacturing factories. The purpose of the present study was to estimate the prevalence of respiratory symptoms and respiratory disorders among workers exposed to dust, benzene, toluene, ethyl-benzene, and xylene (BTEX) as well as silica in the automobile manufacturing factories in Tehran, Iran.

## Materials and Methods

Subjects included workers employing at an automobile manufacturing factory in Tehran, Iran. This study was done in 2016. Eighty samples including 40 workers exposed to varying level of BTEX as well as 40 unexposed controls at an automobile manufacturing was considered to be evaluated. In addition, 80 samples (40 exposed workers and 40 controls) were used for respiratory symptoms and lung function evaluations. All participants were working on the day shift. The duration time of exposures to pollutants in the workplace was ranged between 1 to 8 h/day.

Before their participation in the study, informed consent form was taken from the participants.

### Sampling and analysis of BTEX

Sampling and analysis of BTEX compounds in air inhaled were carried out using NIOSH method number of 1501. Overall, 80 samples were collected for 8 h during working hour. Air was aspirated at a known flow rate through the sampling tubes, containing activated coconut shell charcoal to collect air samples in the workplace. Battery, operated low volume samplers, were used for collecting samples at flow rate of 100 ml/min. Pumps having stable low flow rates (10 to 200 ml/min) were preferable for long period sampling (up to 8 h) or when the concentration of organic vapors is expected to be high. Low volumes of air sample prevented exceeding the adsorptive capacity of the charcoal tubes.

After collection, the compounds were extracted with CS_2_ (2 ml). Analyses of samples were performed by using a VARIAN c-3800 gas chromatograph (GC) coupled with an FID. The maximum concentration of benzene, toluene, ethyl-benzene and xylene in the working environment, 1.7, 8.7, 62 and 74 ppm, respectively. Workers exposed to VOCs in automobile manufacturing factory with duration history of exposure from 2 to 16 yr.

### Air sampling and exposure ranking

Concentrations of total dust in the air inhaled were determined with personal air samplers. Forty samples including 20 workers exposed to varying level of total dust as well as 20 unexposed controls were collected for 8 h during working hours. Closed face filter holders were used (Millipore, Massachusetts, and the USA) and particles were collected by Poly Vinyl Chloride (PVC) filter at sampling flow rate of 2 L/min^−1^. The mean concentrations of dust in the inhaled air were determined to calculate increased mass in the filter weight. Concentration of total dust is equal to the sum of respirable dust (diameter less than 5 microns) and inhalable dust (diameter equal or greater of 5 microns).

### Sampling and analysis of silica

Personal air samplers determined concentrations of silica in the inhaled air. Six samples were collected for 8 h during the working hour. Particle collected by silica Mixed Cellulose Ester (MCE) Membrane Filters sampling at flow rate of 2L/min^−1^. Silica analyses were performed by using visible absorption spectrophotometry.

### Pulmonary function tests

Eighty spirometry tests were performed for 40 pollutants-exposed workers and 40 controls. The lung function test was obtained for exposed and non-exposed personal using spirometry and a disposable mouthpiece filter and nose clip during the test. Spirometry tests were performed before the work shift and after two days away from work. The lung function test includes vital capacity (VC), forced vital capacity (FVC), forced expiratory volume in one second (FEV1), FEV1/FVC ratio, the FEF mid quartile average flow (FEF_25–75_), FEV1/VC ratio, and peak expiratory flow (PEF). Finally, the respiratory capacities of pollutants-exposed workers were compared to the control groups.

### Questionnaire

Assessment of respiratory symptom, nonspecific symptoms, and exposure to tobacco smoke was done by using the original questionnaire. This questionnaire includes items on prevalence of respiratory symptom (cough, phlegm, dyspnea, and wheezing), nonspecific symptoms (watery nose, dry throat, headache, and fatigue), respiratory diseases (sinusitis and asthma) and respiratory illness in the automobile manufacturing factories workers in Tehran.

### Statistics analysis

The data were analyzed by using the statistical package for social science SPSS (ver. 22, Chicago, IL, USA). Comparison between the mean of pollutants concentrations in personnel and lung function measurements was used by one way ANOVA test and multi-regression analysis to determine the relation the between parameter. A two-tailed χ^2^ test of independence was used to determine whether the test item had any significant association with respiratory symptoms between pollutants-exposed workers and control groups.

## Results

### History of workers and control

Overall, 40 men Pollutants-exposed workers, age 25 to 54 yr (mean 34.22 ± 6.85), exposed over periods of 2 to 16 yr (mean 6.9 ± 4.13), had worked 8–10 h/day, were compared to 40 men unexposed, age 36 to 52 (mean 44.5 ± 4.27), in the automobile manufacturing factories in Tehran, Iran. The control group was not exposed to any pollutant of this study interest. The information from workers and control group are summarized in Tables 1 and 2.

**Table 1:** Demographic data of workers (n=40)

***Variable***		***Age groups (yr)***				***Experience groups (yr)***				
		**20–29**	**30–39**	**40–49**	**>50**	**<5**	**5–10**	**10–15**	**15–20**	**>20**
Frequency, n (%)		11 (27.5)	17 (42.5)	11 (27.5)	1 (2.5)	18 (45)	14 (35)	7 (17.5)	1 (2.5)	0 (0)
BMI (kg/m^2^), (mean ± SD)		26.01±3.6	24.73±3.1	26.91±5.02	23.3	24.8±3.5	24.96±3.7	29.05±3.7	25.06	0 (0)
Marital status, n (%)	Yes	9 (22.5)	16 (40)	11 (27.5)	1 (2.5)	15 (37.5)	14 (35)	7 (17.5)	1 (2.5)	0 (0)
	NO	2 (5)	1 (2.5)	0 (0)	0 (0)	3 (7.5)	0 (0)	0 (0)	0 (0)	0 (0)
Smoking, n (%)	Yes	5(12.5)	6(15)	4(10)	0 (0)	9(22.5)	5(12.5)	0 (0)	1 (2.5)	0 (0)
	NO	6(15)	11(27.5)	11(27.5)	1 (2.5)	9(22.5)	9(22.5)	7(17.5)	0 (0)	0 (0)

**Table 2:** Demographic data of control group (n=40)

***Variable***		***Age groups (yr)***				***Experience groups (yr)***				
		**20–29**	**30–39**	**40–49**	**>50**	**<5**	**5–10**	**10–15**	**15–20**	**>20**
Frequency, n (%)		0 (0)	5 (12.5)	31 (77.5)	4 (10)	0 (0)	1 (2.5)	16 (40)	18 (45)	5 (12.5)
BMI (kg/m^2^), (mean ± SD)		0 (0)	25.56±3.8	27.53±3.9	28.42±4.5	0 (0)	30.42	26.96±2.77	27.64±4.6	27.1±5.0
Marital status, n (%)	Yes	0 (0)	5 (12.5)	31 (77.5)	4 (10)	0 (0)	1 (2.5)	16 (40)	18 (45)	5 (12.5)
	NO	0 (0)	0 (0)	0 (0)	0 (0)	0 (0)	0 (0)	0 (0)	0 (0)	0 (0)
Smoking, n (%)	Yes	0 (0)	1 (2.5)	10 (25)	2 (5)	0 (0)	1 (2.5)	4 (10)	5 (12.5)	3 (7.5)
	NO	0 (0)	4 (10)	21(52.5)	2 (5)	0 (0)	0 (0)	12 (30)	13 (32.5)	2 (5)

### Personal air BTEX

Overall, 80 samples were collected during the campaign. The 4-wk average of benzene, toluene, ethyl-benzene, and xylene exposure levels in exposed subjects were 0.775±0.12, 1.2±2.08, 45.8±8.5 and 42.5±23.9 ppm, respectively (Table 3). BTEX compounds were not detected in the breathing zone of control workers.

**Table 3:** Exposure levels of BTEX in exposed workers in Tehran, Iran

	***Benzene***		***Toluene***		***Ethylbenzene***		***Xylene***	
	***Median***	***Range***	***Median***	***Range***	***Median***	***Range***	***Median***	***Range***
Current day levels (ppm)	0.69±0.14	0.54 – 0.83	2.29±3.2	0.4 – 6.08	40.6±2.08	39 – 43	35.1±27.6	4.05 – 57
Four-week average (ppm)	0.775±0.12	0.54 – 0.86	1.2±2.08	0 – 6.08	45.8±8.5	39 – 60	42.5±23.9	4.05 – 67
Cumulative (ppm-years)	0.51±0.42	0.001 – 1.7	1.52±2.55	0 – 8.7	50.4±10.5	39 – 62	51.3±24.73	4.05 – 74

Comparing concentrations of BTEX to recommended standard level showed that, concentration of benzene in the breathing zone of pollutants-exposed workers were significantly higher than TLV-TWA recommended by ACGIH (*P*<0.05). The concentrations of toluene, ethyl-benzene, and xylene were below the standard level recommended by ACGIH.

### Dust exposure

The threshold limit value (TLV) dust exposure content in air is 10 mg.m^−3^ (24). The dust means level and standard deviation of pollutant-exposed was 8.7±2.68 mg.m^−3^ having a range of 5 to 14.4 mg.m^−3^ (Table 4). The dust means level and standard deviation of controls workers was 0. 4±0.013 mg.m^−3^.

**Table 4:** Exposure levels of inspirable dust concentrations in exposed workers in Tehran, Iran

***Concentration***	***Samples (n)***	***Inspirable particulate mass mg.m^−3^***	
		***Mean±SD***	***Range***
Current day levels (mg.m^−3^)	4	7.6±1.2	9.1–6.3
Four-week average (mg.m^−3^)	7	7.3±1.04	8.5–5.7
Cumulative (mg.m^−3^-years)	20	8.7±2.68	14.4–5

### Silica exposure

[Threshold limit value (TLV) for silica of 0.025 mg.m^−3^ was accepted in 2006 by the American Conference of Governmental Industrial Hygienists (ACGIH, 2006)] (25). The free silica mean level and standard deviation of pollutants-exposed was 0.017±0.02 mg.m^−3^ having a range of 0.007 to 0.06 mg.m^−3^.

### Respiratory symptoms

Table 5 presents the prevalence of chronic respiratory symptoms for workers of two groups, including pollutant-exposed workers and control group. Symptoms show a statistically significant difference prevalence of chronic cough and/or phlegm, wheezing, dyspnea, chest tightness, dry throat, headache, fatigue, and running nose in pollutants-exposed workers compare to the control group. Occupational asthma does not report by of two groups of pollutants-exposed workers and control group.

**Table 5:** Respiratory symptoms in 80 samples (40 exposed workers and 40 controls)

***Symptom***	***Case participant***	***Control participant***	***P-value***
Dry throat	14 (35)	1 (2.5)	0.00001
Running nose	14 (35)	1 (2.5)	0.00001
Headache	10 (25)	3 (7.5)	0.03
Fatigue	17 (42.5)	3 (7.5)	0.001
Sinusitis	4 (10)	1 (2.5)	0.1
cough	20 (50)	5 (12.5)	0.001
Phlegm	19 (47.5)	5 (12.5)	0.001
Wheezing	22 (55)	5 (12.5)	0.0001
Dyspnea	17 (42.5)	3 (7.5)	0.001
Chest tightness	14 (35)	3 (7.5)	0.003

### Airway responsiveness

Table 6 shows the average change in lung function measurements before and after being employed in profession. Statistical tests show significant difference between pulmonary function tests (except PEF) of exposed and non-exposed personal before and after employment in job.

**Table 6:** Lung function measurements before and after employment in 80 samples (40 exposed workers and 40 controls)

***Parameter***	***Before employment***		***After employment***		***P-value***
	***Case participant***	***Control participant***	***Case participant***	***Control participant***	
FVC (L)	5.02±0.61	4.85±0.52	4.69±0.65	4.80±0.49	0.0001
FEV1 (L)	4.16±0.50	4.02±0.40	3.84±0.51	3.80±0.46	0.0001
FEV1/FVC%	82.61±4.07	81.94±2.99	81.12±6.64	79.39±3.26	0.002
PEF (L/S)	9.25±1.15	9.09±1.07	8.74±1.45	9.07±1.51	0.071
FEF2575 (L/S)	4.58±0.80	4.33±0.52	3.91±0.84	3.67±0.63	0.0001
VC (L)	4.89±0.61	4.77±0.52	4.67±0.70	4.75±0.55	0.016
FEV1/VC(%)	83.36±13.54	83.98±4.01	82.53±6.4	80.0±4.40	0.034

Workers exposed to pollutants have presented lower levels of FVC, VC, and PEF compare to the control group except FEV1/FVC%, FEV1, FEF2575 and FEV1/VC% (Table 7).

**Table 7:** Lung function parameters among parameters in 80 samples (40 exposed workers and 40 controls)

***Parameter***	***Case participant (n=40)***		***Control participant (n=40)***		***P-value***
	***Smoke (n=15)***	***Non-smoke (n=25)***	***Smoke (n=13)***	***Non-smoke (n=27)***	
FVC (L)	4.93±0.44	4.55±0.72	4.44±0.43	4.97±0.42	0.41
FEV1 (L)	4.01±0.41	3.74±0.55	3.44±0.44	3.97±0.36	0.69
FEV1/FVC (%)	78.38±6.81	82.77±6.08	78.4±4.98	79.87±1.93	0.14
PEF (L/S)	9.02±1.47	8.58±1.45	7.72±1.89	9.72±0.66	0.32
FEF2575 (L/S)	3.95±0.77	3.89±0.89	3.36±0.71	3.82±0.54	0.15
VC (L)	4.92±0.50	4.52±0.76	4.42±0.42	4.91±0.54	0.57
FEV1/VC (%)	81.18±6.50	83.34±6.33	78.61±6.39	80.67±2.96	0.04

In the study groups, the FVC, VC, and PEF were significantly reduced, however, FEV/FVC, FEV1, FEF2575 and FEV1/VC did not show significant difference between pulmonary function tests of exposed and non-exposed person. In this investigation, person smoking was significantly associated with FEV1/FVC and PEF (*P*<0.05) (Table 8).

**Table 8:** Relationship between each respiratory function parameters in 40 Pollutants-exposed workers and age, duration of employment, smoking, and BMI

***Parameter***	***Age (yr) correlation***	***Duration of employment (years) correlation***	***Smoking (yes /no) t-test***	***BMI correlation***
FVC (L)	*P*=0.015	*P*=0.84	*P*=0.6	*P*=0.1
FEV1 (L)	*P*=0.001	*P*=0.15	*P*=0.3	*P*=0.03
FEV1/FVC (%)	*P*=0.38	*P*=0.03	*P*=0.01	*P*=0.8
PEF (L/S)	*P*=0.40	*P*=0.96	*P*=0.03	*P*=0.8
FEF2575 (L/S)	*P*=0.005	*P*=0.02	*P*=0.3	*P*=0.5
VC (L)	*P*=0.009	*P*=0.6	*P*=0.8	*P*=0.1
FEV1/VC (%)	*P*=0.213	*P*=0.02	*P*=0.1	*P*=0.5

The information of lung function parameters in case and control groups with duration of employment have been summarized in Table 9.

**Table 9:** Distribution of lung function test parameters in 40 pollutants-exposed workers and 40 controls considering duration of employment (years)

***Parameter***	***Case participant (n=40)***			***Control participant (n=40)***			**P-value**
	**≥10(n=32)**	**10–19(n=8)**	**≥20(n0)**	**≥10(n=1)**	**10–19(n=33)**	**≥20(n=6)**	
FVC (L)	4.72±0.65	4.56±0.65	0	4.58	4.79±0.47	4.88±0.68	0.84
FEV1 (L)	3.88±0.48	3.68±0.63	0	3.03	3.84±0.40	3.7±0.70	0.15
FEV1/FVC (%)	81.37±7.26	80.15±3.31	0	79	80.09±2.52	75.63±4.68	0.03
PEF (L/S)	8.75±1.47	8.71±1.49	0	9.22	9.24±1.22	8.13±2.66	0.96
FEF2575 (L/S)	3.98±0.80	3.63±0.98	0	3.42	3.74±0.60	3.35±0.81	0.02
VC (L)	4.72±0.70	4.48±0.68	0	4.58	4.74±0.54	4.88±0.68	0.6
FEV1/VC (%)	82.63±6.77	82.11±5.01	0	79	80.87±3.73	75.36±5.57	0.02

## Discussion

Workers are potentially exposed to biological pollutants, chemical airborne, and dust resulting in adverse effect on their health (13, 18). Strength of this study, which helped in declining bias related to the sampling and data collection, is that lung function, chronic respiratory symptoms, and analysis of data were carried out by the same researcher.

Work in automobile manufacturing factories may cause the increase in chronic respiratory symptoms accompanied by deterioration in lung function in pollutant-exposed workers. These changes are particularly pronounced in those workers exposed to the dust, BTEX, and silica. Since the respiratory symptoms are self-reported, workers exposed to the pollutants could have over-reported symptoms and controls participant could have under-reported symptoms.

Self-reported workplace exposure has demonstrated more consistent association with high risks of lung disorders as well as respiratory symptoms (26–28). However, the value of research questionnaire depends on the regional culture (29). According to this study, a significantly higher prevalence of respiratory symptoms in pollutant-exposed workers was found. In this study, cough, phlegm, and wheezing were in higher prevalence, while Sinusitis and Headache were in lower prevalence of respiratory symptom in pollutant-exposed workers. In flour mill workers, cough and chronic expectoration were in higher prevalence (30). Prevalence of respiratory symptom in the pollutant exposed workers probable cause increased risk of respiratory disease and initiation of new symptoms (7).

Occupational asthma was not reported by of two groups. Occupational asthma was in lower prevalence in confectionery workers (31). 29% of the population was exposed to dust or gas, 5% exposed to asbestos at work and 4% was reported exposure to quartz. Occupational dust or gas exposure was associated with a chronic cough, phlegm when coughing, breathlessness on exercise, and occasional wheezing (32). High risk of adult-onset respiratory symptoms was reported among mechanics, repairers, cleaning, and building service workers. Moreover, increases in wheezing (4%-21%) and shortness of breath (4%–8%) among Latino farmworkers engaged in crop production (33).

Measurement of lung function is more reliable than a questionnaire for detecting chronic obstructive pulmonary disease (34, 35). Workers exposed to pollutant had significantly (*P*≥0.05) lower levels of FVC, VC, and PEF than the control group, except FEV1/FVC%, FEV1, FEF2575 and FEV1/VC% as shown in Table 7. The reason behind the higher FEV1/FVC the control group is due to the increased FEV1. Workers exposed to dust had lower levels of FVC, VC, and PEF than the control group (36–39). Respiratory capacity of employees may be changed due to the high concentration of dust. Moreover, he/she reported a decreased in the peak flow rate (PFR), forced expiratory volume (FEV %) (40).

Decline in lung volumes and respiratory symptoms were significantly associated with the exposure to dust or gas, duration of exposure, and smoke. Lung function tests should be performed before and after the employment by measures of lung function to identify sensitive workers. The respiratory questionnaire is recommended to be used before starting employment for identifies sensitive workers in the pollutant workplace. Those with pre-existing lung disease and smokers may be at the particular risk for working in those areas of automobile manufacturing factories that induce respiratory and acute or chronic respiratory disease.

## Conclusion

Workers exposed to air contaminations including dust, BTEX, and silica in the factories, show considerable respiratory disorders as well as adverse effects on alveolar airways in lungs compared to the control group.

## Ethical considerations

Ethical issues (Including plagiarism, informed consent, misconduct, data fabrication and/or falsification, double publication and/or submission, redundancy, etc.) have been completely observed by the authors.
